# Predicting and Avoiding Complications in Percutaneous Nephrolithotomy in the Era of Personalized Medicine: A Scoping Review

**DOI:** 10.3390/jpm14090962

**Published:** 2024-09-10

**Authors:** Carlos Fernández Baltar, María Elena Martínez Corral, Daniel Pérez Fentes

**Affiliations:** 1 Department of Urology, University Hospital Complex of Pontevedra, 36071 Pontevedra, Spain; 2 Department of Urology, University Hospital Complex of Santiago de Compostela, 15706 Santiago de Compostela, Spain; maria.elena.martinez.corral@sergas.es (M.E.M.C.); daniel.adolfo.perez.fentes@sergas.es (D.P.F.)

**Keywords:** percutaneous nephrolithotomy, PCNL, complications, prediction, prevention, technology, artificial intelligence, 3D models, image guidance, biomarkers

## Abstract

Background: Percutaneous nephrolithotomy (PCNL) is associated with a wide range of complications. This review aims to explore how recent technological advancements and personalized medicine can help prevent or predict these complications. Methods: A scoping review was conducted according to the PRISMA-SCR guidelines and registered on the Open Science Framework in April 2024. A literature search was performed on PUBMED, Web of Science, and Scopus databases. This review focused on predictive AI models, 3D surgical models, intrasurgical image guidance, and biomarkers. Articles meeting the following criteria were included: publication between 2019 and 2024, written in English, involving human participants, and discussing technological advancements or personalized medicine in the context of complications in PCNL. Results: Of the 11,098 articles searched, 35 new studies were included. We identified a few articles on predictive AI models. Several studies demonstrated that 3D presurgical models and virtual models could enhance surgical planning and reduce complications. New intrasurgical image and guidance systems showed the potential in reducing bleeding and radiation exposure. Finally, several biomarkers were identified as predictors of sepsis and other complications. Conclusion: This scoping review highlights the potential of emerging technologies in reducing and predicting PCNL complications. However, larger prospective studies are required for validation.

## 1. Introduction

The total complication rate in percutaneous nephrolithotomy (PCNL) varies across studies, with recent reviews indicating rates between 4% and 50.8% [1]. In the work by La Rosette et al., which presents multicentre results from the Clinical Research Office of the Endourological Society (CROES) working group, an incidence of complications of 20.5% is reported [2]. For systematic analysis, PCNL complications can be categorized into three main groups: haemorrhagic, infectious, and miscellaneous.

Among the first group of complications, we encounter postoperative bleeding in its various manifestations, which is common following PCNL. It usually manifests as mild to moderate non-anaemic postoperative haematuria that typically resolves without intervention. Occasionally, small perirenal hematomas may develop, affecting more than one-third of patients [3]. The transfusion rate attributed to PCNL-related bleeding stands at approximately 7%, with reported rates ranging between 0.43% and 20%. Noteworthy risk factors include the presence of multiple tracts, renal pelvis perforation, procedural inexperience, and preoperative anaemia [4,5,6]. The need for embolization due to bleeding ranges from 0.8% to 1.3% [7,8]. It is generally accepted that bleeding occurring during or immediately after PCNL commonly arises from venous bleeding or injuries to the segmental or interlobar arteries, which are often sustained during puncture and tract dilation or due to excessive angulation of endoscopic instruments. Key techniques to mitigate bleeding risk include respecting renal vascular anatomy, performing peripheral puncture through the calyx papilla, and exercising caution during dilation within the infundibulum [9,10].

In the second group of complications, we find infectious complications, which can vary in severity from the onset of postoperative fever to a case of septic shock. Postoperative fever occurs in 21% to 32% of patients, making it one of the most common complications. Most infectious complications have a good outcome if diagnosed and treated early. Nevertheless, between 0.3% and 4.7% of cases [11] progress to sepsis in different degrees, with published mortality rates of up to 66% [12], making it one of the main causes of mortality associated with PCNL. Although the appearance of infectious complications is related to the manipulation of the lithiasis itself (often colonized by bacteria), certain measures can help reduce their incidence. It is essential to ensure that the patient has sterile urine prior to the intervention through urine cultures and perform adequate antibiotic prophylaxis even in patients with negative urine cultures [13,14,15]. The kidney must be properly drained before the intervention to avoid the presence of pyonephrosis. Intraoperative measures that have been shown to decrease the occurrence of infectious complications include avoiding hyperpressure in the tract and maintaining reasonable surgical times (below 90–120 min) [16,17].

Lastly, there is a miscellany of complications secondary to the puncture of adjacent structures. Pneumothorax, hydrothorax, or hemothorax can occur, with a much higher risk if the puncture is made between the tenth and eleventh ribs. The use of ultrasound for puncture guidance and the performance of the procedure during expiration decrease the risk of pleural puncture. Perforation of the pyelocaliceal system during PCNL can lead to reabsorption syndrome, which increases with surgical time and, consequently, with irrigated volume. Perforation of hollow viscera, as well as hepatic and splenic damage, can occur in up to 1% of cases (especially if not guided via ultrasound), being more frequent in horseshoe kidneys, patients with neurological damage, or those with previous interventions.

In recent years, numerous technological advancements have emerged, which could aid in preventing or predicting PCNL-related complications. Various examples of innovations, such as 3D models, augmented reality devices for intraoperative assistance, novel biomarkers, or the unstoppable and emerging use of artificial intelligence, are technologies that have the potential to revolutionize the field of endourology. A scoping review was conducted to systematically map the research in this area and identify existing knowledge gaps. The following research questions were formulated: Can the utilization of new technologies prevent complications in percutaneous nephrolithotomy? And how can personalized medicine predict the occurrence of complications in patients undergoing percutaneous nephrolithotomy?

## 2. Materials and Methods

### 2.1. Protocol and Registration

The protocol was drafted using the Preferred Reporting Items for Systematic Reviews and Meta-analysis for Scoping Reviews (PRISMA-SCR), and it was registered in the open Science Framework on 21 April 2024. The PRISMA-SCR checklist is available in Appendix A.

### 2.2. Search Strategy

In April 2024, a literature search on PUBMED, Web of Science, and Scopus databases was conducted to locate articles related to the application of new technologies or personalized medicine and the occurrence of complications in percutaneous nephrolithotomy.

The keywords used in our search strategy were as follows: percutaneous nephrolithotomy and complications, haemorrhagic, sepsis, infection, artificial intelligence (A.I), neural network, machine learning, puncture, access, robotics, biomarkers, prediction, genetics, genomics. A detailed search strategy is available in Appendix B. Two investigators conducted an initial screening based on titles and abstracts to identify eligible studies. Potentially relevant studies underwent a full-text review. Moreover, manual searches of reference lists of relevant articles were performed to identify additional studies. Any disagreements were resolved through consensus among co-authors.

### 2.3. Inclusion and Exclusion Criteria

Peer-reviewed journal articles meeting the following criteria were included: publication between 2019 and 2024, written in English, involving human participants in the context of technological advancements or personalized medicine, and the occurrence of complications in percutaneous nephrolithotomy. Articles that did not align with the study’s conceptual framework were excluded, such as those focusing on the prediction of complications using traditional variables (stone burden, preoperative culture, time, etc.) or technological advancements unrelated to the occurrence of complications.

### 2.4. Synthesis of Results

We grouped the studies according to the type of technology and the surgical phase they are aimed towards preventing or predicting: predictive A.I. models, 3D surgical models, intrasurgical image guidance, and biomarkers. A brief narrative synthesis of the included studies has been conducted, detailing the type of technology used and the actual or expected benefits in relation to complications. Tables were created for evidence synthesis, including sample size, study type, type of technology, and the relationship with complication reduction. Quantitative data were not included for the latter parameter due to the heterogeneity of the included studies.

## 3. Results

**Figure 1:**
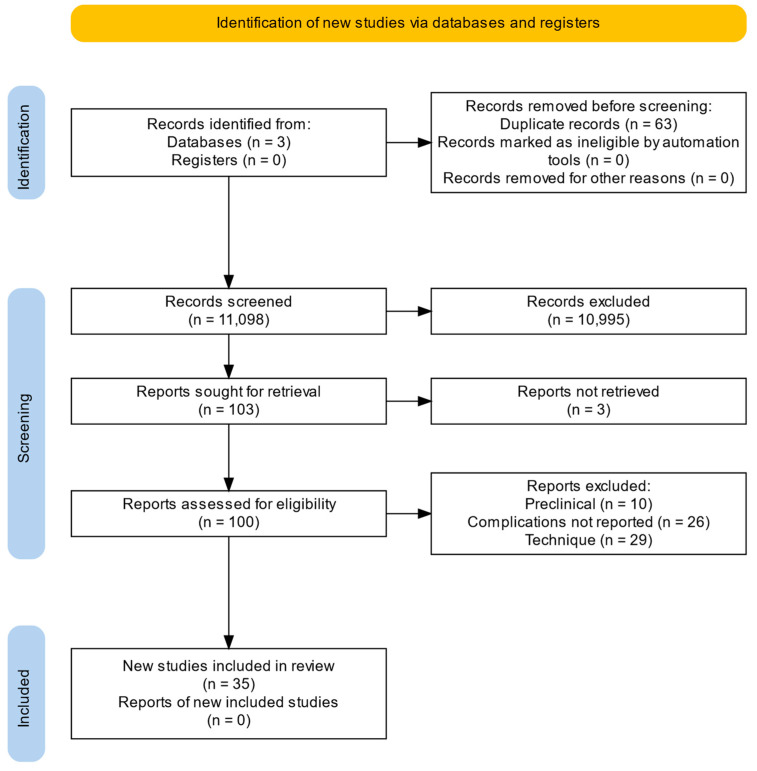
PRISMA flowchart.

Of the 11,098 articles searched, 100 met the eligibility criteria and were included for full-text reviews. In total, 35 new studies were included in the scoping review. Figure 1 shows the PRISMA flowchart for study identification and the selection of outcomes.

### 3.1. Predictive A.I. Models

Geragthy et al. [18] developed a predictive model using data from a large national prospective database (British Association of Urological Surgeons PCNL, *n* = 12,810). They combined machine learning techniques, including extreme gradient boosting and deep neural networks, with logistic regression. This model can predict nine outcomes (visceral injury, need for transfusion, postoperative infection, postoperative complications, need for higher care, immediate clearance on intraoperative fluoroscopy, clearance on immediate postoperative imaging, stone-free status at follow-up, and need for adjuvant treatment) from eleven input parameters and is accessible as an online tool. The predictive capacity using ROC curves was between 0.59 and 0.94 depending on the outcome evaluated.

Alexander Izrailevich et al. [19] created a neural-network-based tool using retrospective database analysis to customize treatment options (shock wave lithotripsy, PCNL, or pyelolithotomy), aiming to minimize the risk of postoperative complications. Using this tool to choose the optimal treatment method, they report higher ESWL efficiency in the experimental group due to increased stone fragmentation, with lower energy costs and fewer sessions.

Meng et al. [20] developed a machine learning-based predictive model for bleeding after lateral decubitus PCNL, based on a retrospective study of 356 patients, achieving an area under curve (AUC) of 0.679. Similarly, Shen et al. used machine learning to evaluate the prediction of postoperative sepsis in a study with 694 patients, achieving an AUC of 0.89.

### 3.2. Three-Dimensional Presurgical Models

Cui et al. [21] conducted a prospective randomized study involving 45 patients in each arm to investigate the utility of a preoperative 3D-printed model for surgical planning. The study reported a higher stone-free rate (96% in the model group vs. 80% in the control group) and a lower complication rate (6.67% vs. 22.2%; *p* = 0.02). 

Ghazi et al. [22], in a prospective study involving a fellowship-trained endourologist performing 20 consecutive procedures, compared the first 10 standard procedures with the next 10 procedures pre-simulated using a 3D hydrogel model. The latter group had fewer complications (1 vs. 5; *p* < 0.001) and other improved parameters (mean fluoroscopy time, percutaneous needle access attempts, complications, and additional procedures). Conversely, Liu et al. [23] found an improvement in stone-free rates but not in complication rates in a similar study using a 3D-printed model.

Other studies have evaluated the utility of virtual 3D models. Tan et al. [24], in a retrospective study of 139 patients, used virtual 3D reconstructions prior to PCNL and compared their results with a non-reconstruction group, reporting significantly fewer complications in the 3D reconstruction group (8.3% vs. 25.4%). 

Huang et al. [25] and Zhu et al. [26] found that the outcomes of a 3D virtual model with simulated punctures diminished operation times, the number of punctures, and intraoperative blood loss. However, Hosseini et al. [27], in a prospective randomized study of 48 patients using a virtual 3D model, demonstrated a reduction in the number of puncture attempts and radiation exposure but did not find a significant reduction in complication rates. Similar results were reported in Qin’s study [28].

Using a different approach, Keyu et al. [29] conducted a randomized study with 22 patients using a 3D-printed personalized percutaneous puncture guide access plate. This method achieved a 100% first-attempt access rate and reduced times compared to the standard access control group, resulting in less bleeding (49 mL vs. 60 mL), although they did not measure a clinical reduction in complications.

Regarding prediction, Özbir et al. [30] used 3D volumetry to create a ratio between the segmentation of the renal collecting system volume (RCSV), reflecting the distribution of the stone burden volume in the pelvicalyceal system, and they analysed the stone volume (ASV). They correlated this ratio with the occurrence of complications using ROC curves (AUC of 0.869; *p* < 0.001, sensitivity of 93.3%, specificity of 78.1%).

Table 1 shows a summary of the relationship between different 3D presurgical models and the appearance of complications.

### 3.3. Intrasurgical Image and Guidance Systems

Rassweiler-Seyfried et al. [31] explored augmented reality by performing an iPad-assisted puncture in 22 patients, finding significant differences only in reducing radiation exposure and puncture time in a matched pair analysis. Also, Porpiglia et al. [32] 21 investigated augmented reality with 3D mixed-reality holograms compared to a retrospective cohort. This study reported no major complications and reduced radiation exposure, but at the expense of longer puncture times. In Wang’s study [33], the same technology was used, and although the experimental group had fewer complications, the difference was not statistically significant.

Several studies have explored new ultrasound techniques, including contrast-enhanced ultrasound [34,35,36,37] or colour Doppler ultrasound targeting avascular areas [38] for puncture guidance. These studies are summarized in Table 2.

In Jiao’s study [39], CT-guided 3D virtual navigation puncture was utilized without serious complications in patients who could not be accessed using standard techniques. Taguchi et al. [40] studied the performance of robotic-assisted fluoroscopic-guided punctures but did not find a reduction in complications.

Table 2 shows a summary of the relationship between different intrasurgical image and guidance systems and the appearance of complications.

### 3.4. Biomarkers

Ahmed et al. [41] conducted a study on stone and midstream urine samples, finding a relationship between antibiotic resistance and virulence genes and proposing stone culture as a predictor of postoperative septic complications.

Several novel candidates have been proposed as predictive postoperative biomarkers. The NOD2 gene was identified as a postsurgical predictor of sepsis [42,43]. Low CD3+ cell/high-IL2r has been suggested as a predictor for systemic inflammatory response syndrome (SIRS) [44]. Procalcitonin [45,46] and IL-6 [47] have also been demonstrated to predict sepsis. A relation between neutrophil–lymphocyte ratio (NLR), platelet–lymphocyte ratio (PLR), and lymphocyte–monocyte ratio (LMR) and sepsis was suggested in two recent studies [48,49]. Finally, the albumin–globulin ratio (AGR) and the high-sensitivity C-reactive protein/albumin ratio have been associated with SIRS [50,51].

Table 3 shows a summary of the relationship between different biomarkers and their ability to predict postoperative fever, SIRS, or sepsis.

## 4. Discussion

Scoping reviews aim to comprehensively examine the literature on broader questions compared to other types of reviews [52]. In our case, we explored whether new technologies could reduce or predict complications in PCNL.

Regarding the reporting of complications, standardization through the use of the Clavien–Dindo classification is crucial. We were unable to include this classification in the review because the vast majority of studies did not provide this information. We firmly believe that any surgical outcome in endourology should include these data as the only way to ensure the comparability of results.

Artificial intelligence (AI) is one technology with the potential to revolutionize medicine. However, we identified only three articles on AI applied to predictive models for complications in PCNL, none of which included external validation. Additionally, no studies utilized radiomics in this regard. We believe that the use of artificial intelligence for predicting complications holds incredible potential, allowing for preventive measures to be taken at all levels. Although the reviewed studies are limited, they open new avenues for research. We could classify and identify the most complex cases to plan them more appropriately preoperatively. On the other hand, predictive systems for the onset of sepsis could be developed both pre- and postoperatively, enabling early intervention and reducing the severity of complications.

In endourology, particularly PCNL, complication rates are influenced by annual case volumes at the centre [53] and the effect of the learning curve [54,55]. Implementing assistive technologies could mitigate this learning curve and facilitate the adoption of PCNL in lower-volume centres. Although not covered in this study, training residents and fellows using simulation in virtual platforms and 3D-printed models [56,57] could be an essential initial step in making the learning curve smoother, despite the challenges in measuring real-life outcomes.

A critical aspect of PCNL is respecting renal anatomy and achieving access via a transpapillary puncture [9,10], a concept supported by traditional studies but recently challenged by new research [58,59]. We identified several studies that suggest creating pre-surgical virtual and 3D-printed models to meticulously plan puncture trajectories and simulate lithotripsy. While some studies indicate a trend towards reduced complications, their clinical relevance appears limited due to small sample sizes and the lack of randomization. Alternatively, in the case of 3D models, a significant limitation to consider is the anatomical alteration resulting from changes in position (prone or supine) and subsequently from the dilation of renal cavities due to the infusion of fluid or contrast.

We also reviewed studies on intraoperative assistance, including augmented reality, enhanced ultrasound techniques, and robotic puncture. Although most studies are small, some show a trend towards reduced bleeding. Emerging technologies not included in this review, such as Automated Needle Targeting with X-ray (ANT-X) [60] and 5G teleassistance during surgery, demonstrate promising potential. However, challenges remain, as evidenced by Spenkelin’s study on stereotactic optical navigation, which was prematurely closed due to low technical success. Preliminary reports also exist with respect to other guidance technologies [61,62,63]. Remarkably, we have not identified any studies relating the type of lithotripsy energy used (laser, ultrasonic, or ballistic) to the occurrence of complications.

One of the most serious complications in PCNL is sepsis, a leading cause of mortality associated with the procedure. Early detection and intervention are crucial for improving survival rates [64]. We sought studies on new postoperative biomarkers for sepsis or the validation of existing ones. Despite the critical importance of this area, we found few studies, most of which were retrospective and involved small patient populations. While these studies primarily focus on biomarkers derived from the body’s immune and inflammatory response, future research should also consider bacterial virulence factors in the lithic microbiome [41]. Notably, we did not identify any significant studies on the genetics and genomics of PCNL complications.

We have deliberately not included studies related to miniaturization and aspiration sheaths in this technological review, as they are already well documented in the literature and widely implemented in most endourology services [65,66,67]. However, we would like to briefly address this topic, as it serves as a strong example of how technology can directly impact the reduction in complications in percutaneous surgery. Studies indicate that using miniaturized calibers (sheaths < 20 Ch) tends to reduce complications, namely hemorrhagic ones, by causing less damage to the renal parenchyma. However, this benefit may come at the cost of longer surgical times, depending on the stone burden being treated. There is also ongoing controversy about whether miniaturization reduces or potentially increases infectious complications. Infection with these systems may be due to two factors: with some systems, high intrarenal pressure can be created, and prolonged surgical times can also contribute to infection risk. More recently, the use of aspiration sheaths has been introduced with two main objectives: to facilitate the removal of stone fragments, shortening surgical times and enhancing complete stone clearance, and reduce intrarenal pressure through aspiration, which can decrease the occurrence of complications. By preventing intrarenal pressure from exceeding 40 mmHg, these sheaths may reduce postoperative pain and pyrexia. Nonetheless, further randomized in vivo studies are needed to confirm this hypothesis.

## 5. Study Limitations

Our scoping review has some limitations. In an attempt to focus only on advances in technology, we have limited the literature search to the last 5 years. On the other hand, we have carried out a complication-centred search; we are aware that new technologies often take time to demonstrate their usefulness in this sense.

## 6. Conclusions

Recent technological advancements hold significant potential for reducing and predicting complications in PCNL. However, larger prospective studies are essential for the proper validation of these innovations. There is a clear need for further research in genetics, genomics, radiomics, and microbiomics to bridge existing knowledge gaps in these areas.

## Figures and Tables

**Table 1:** Relationship between different 3D presurgical models and complications.

Reference	Sample Size (*n*)	Type of Study	Technology	Diminished Complications
[21]	45	Prospective randomized	Printed 3D model	Yes
[22]	20	Prospective non-randomized	Printed 3D model	Yes
[23]	72	Prospective randomized	Printed 3D model	No
[24]	139	Retrospective	Virtual 3D model	Yes
[25]	120	Prospective randomized	Virtual 3D model	Less intrasurgical bleeding
[26]	140	Prospective randomized	Virtual 3D model	Less Hb drop
[27]	48	Prospective randomized	Virtual 3D model	No
[28]	60	Retrospective	Virtual 3D model	No
[29]	22	Prospective randomized	Printed 3D puncture guide access plate	Less intrasurgical bleeding
[30]	141	Retrospective	3D volume segmentation	No Prediction of complications

Hb: Haemoglobin; 3D: three dimensional.

**Table 2:** Relationship between different intrasurgical image and guidance systems and complications.

Reference, First Author, Date	Sample Size (*n*)	Type of Study	Technology	Diminished Complications
[31]	44	Prospective non-randomized	Augmented reality (iPad-assisted puncture)	No
[32]	10	Prospective non-randomized	Augmented reality (three-dimensional mixed reality holograms)	No
[33]	61	Prospective	Augmented reality (three-dimensional mixed reality holograms)	No
[34]	160	Prospective randomized	Contrast US vs. standard US	Less Hb drop
[35]	72	Prospective randomized	Contrast US vs. standard US	Less Hb drop
[36]	56	Prospective randomized	Contrast US vs. standard US	Less Hb drop
[37]	228	Retrospective	Dopper colour US vs. standard US	Less Hb drop Less transfusion rate Less LOS
[38]	348	Prospective non-randomized	Dopper colour US vs. Standard US	Less tract bleeding Less Hb drop Less LOS
[39]	33	Retrospective	C-arm CT-guided with 3D virtual navigation after standard failed access.	No
[40]	71	Prospective randomized	Robotic-assisted fluoroscopic-guided vs. Standard US	No

Hb: Haemoglobin; LOS: length of stay; CT: computerized tomography; US: ultrasound.

**Table 3:** Relationship between different biomarkers and predictions of complications.

Reference, First Author, Date	Sample Size (*n*)	Type of Study	Biomarker	Prediction of Complications
[41]	200	Prospective non-randomized	Bacterial virulence genes: *hlb, pvl, fnbB, can* and *seb* (stone culture and PCR assay)	Sepsis
[42]	156	Retrospective	NOD2 gen	Sepsis
[43]	387	Prospective non-randomized	HMGB1/HLA-DR	Sepsis
[44]	154	Retrospective	Low CD3+ cell/high IL2r	SIRS
[45]	407	Retrospective	Procalcitonin	Postoperative fever
[46]	356	Retrospective	Procalcitonin	Sepsis
[47]	90	Retrospective	Interleukin 6	Sepsis
[48]	517	Prospective non-randomized	NLR, PLR, LMR	SIRS/Sepsis
[49]	356	Retrospective	NLR, PLR, LMR	SIRS
[50]	354	Retrospective	Preoperative AGR	SIRS
[51]	556	Retrospective	hs-CRP/Alb ratio	SIRS

SIRS: Systemic inflammatory response syndrome; NLR: neutrophil–lymphocyte ratio; PLR: platelet–lymphocyte ratio; LMR: lymphocyte–monocyte ratio; AGR: albumin–globulin ratio; hs-CRP/Alb: high-sensitivity C-reactive protein/albumin.

## Appendix A. Preferred Reporting Items for Systematic Reviews and Meta-Analyses Extension for Scoping Reviews (PRISMA-ScR) Checklist

**SECTION**	**ITEM**	**PRISMA-ScR CHECKLIST ITEM**	**REPORTED ON PAGE**
**TITLE**			
Title	1	Identify the report as a scoping review.	1
**ABSTRACT**			
Structured summary	2	Provide a structured summary that includes (as applicable): background, objectives, eligibility criteria, sources of evidence, charting methods, results, and conclusions that relate to the review questions and objectives.	1
**INTRODUCTION**			
Rationale	3	Describe the rationale for the review in the context of what is already known. Explain why the review questions/objectives lend themselves to a scoping review approach.	2
Objectives	4	Provide an explicit statement of the questions and objectives being addressed with reference to their key elements (e.g., population or participants, concepts, and context) or other relevant key elements used to conceptualize the review questions and/or objectives.	2
**METHODS**			
Protocol and registration	5	Indicate whether a review protocol exists; state if and where it can be accessed (e.g., a Web address); and if available, provide registration information, including the registration number.	2
Eligibility criteria	6	Specify characteristics of the sources of evidence used as eligibility criteria (e.g., years considered, language, and publication status), and provide a rationale.	3
Information sources *	7	Describe all information sources in the search (e.g., databases with dates of coverage and contact with authors to identify additional sources), as well as the date the most recent search was executed.	3
Search	8	Present the full electronic search strategy for at least 1 database, including any limits used, such that it could be repeated.	3
Selection of sources of evidence	9	State the process for selecting sources of evidence (i.e., screening and eligibility) included in the scoping review.	3
Data charting process	10	Describe the methods of charting data from the included sources of evidence (e.g., calibrated forms or forms that have been tested by the team before their use, and whether data charting was done independently or in duplicate) and any processes for obtaining and confirming data from investigators.	3
Data items	11	List and define all variables for which data were sought and any assumptions and simplifications made.	3
Critical appraisal of individual sources of evidence	12	If done, provide a rationale for conducting a critical appraisal of included sources of evidence; describe the methods used and how this information was used in any data synthesis (if appropriate).	3
Synthesis of results	13	Describe the methods of handling and summarizing the data that were charted.	3
**RESULTS**			
Selection of sources of evidence	14	Give numbers of sources of evidence screened, assessed for eligibility, and included in the review, with reasons for exclusions at each stage, ideally using a flow diagram.	3–6
Characteristics of sources of evidence	15	For each source of evidence, present characteristics for which data were charted and provide the citations.	3–6
Critical appraisal within sources of evidence	16	If done, present data on critical appraisal of included sources of evidence (see item 12).	3–6
Results of individual sources of evidence	17	For each included source of evidence, present the relevant data that were charted that relate to the review questions and objectives.	3–6
Synthesis of results	18	Summarize and/or present the charting results as they relate to the review questions and objectives.	3–6
**DISCUSSION**			
Summary of evidence	19	Summarize the main results (including an overview of concepts, themes, and types of evidence available), link to the review questions and objectives, and consider the relevance to key groups.	7–8
Limitations	20	Discuss the limitations of the scoping review process.	8
Conclusions	21	Provide a general interpretation of the results with respect to the review questions and objectives, as well as potential implications and/or next steps.	8
**FUNDING**			
Funding	22	Describe sources of funding for the included sources of evidence, as well as sources of funding for the scoping review. Describe the role of the funders of the scoping review.	8
		From: Tricco et al. [68].	

## Appendix B. Search Strategy

(Percutaneous nephrolithotomy and complications)
Or
(percutaneous nephrolitotomy and haemorhagic)
Or
(percutaneous nephrolitotomy and sepsis)
Or
(percutaneous nephrolitotomy and artificial intelligence)
Or
(percutaneous nephrolitotomy and neural networks)
Or
(percutaneous nephrolitotomy and access)
Or
(percutaneous nephrolitotomy and robotics)
Or
(percutaneous nephrolitotomy and infection)
Or
(percutaneous nephrolitotomy and biomarkers)
Or
(percutaneous nephrolitotomy and prediction)
Or
(percutaneous nephrolitotomy and genetics)
